# Chatbots reduce health-related conspiracy beliefs not because of but despite being perceived as AI

**DOI:** 10.1038/s41598-026-66242-5

**Published:** 2026-08-17

**Authors:** Paul Ballot, Yana van de Sande, Hanna Schraffenberger, Philipp Schmid

**Affiliations:** 1 iHub, Radboud University, Nijmegen, The Netherlands; 2 Centre for Language Studies, Radboud University, Nijmegen, The Netherlands; 3 Institute for Computing and Information Sciences, Radboud University, Nijmegen, The Netherlands; 4 Institute for Planetary Health Behaviour, University of Erfurt, Erfurt, Germany

**Keywords:** Health misinformation, Persuasion, Infodemic, Belief-revision, Debunking, Generative AI, Psychology, Psychology, Science, technology and society

## Abstract

Beliefs in conspiracy theories and their resistance to correction pose critical challenges to public health. A potential countermeasure to this is the use of Large Language Model (LLM) driven debates about conspiracy theories. Yet while this approach yields promising results for general conspiracies, its generalizability to the health domain as well as the underlying mechanism for its effectiveness remain unclear. This study investigates whether LLM-driven debates can reduce health-related conspiracy theories in the context of COVID-19. Furthermore, it examines attribution to artificial rather than human sources as a potential explanation. In an online experiment, 554 participants were randomly assigned to either a control condition or to debate an individual COVID-19 conspiracy theory with an AI-labelled LLM or a human-labelled LLM. Compared to the control, participants aware of their artificial conversation partner reported 7.88% points less confidence in the conspiracy after the intervention (*d* = 0.63, *p* < .001). Contrary to our expectation, however, this effect was stronger for those assuming a human conversation partner: They indicated a 13.76% points larger confidence drop compared to the control (*d* = 0.87, *p* < .001). This difference in effectiveness appears to be mediated by the perceived neutrality of the source.

**Supplementary Information:**

The online version contains supplementary material available at 10.1038/s41598-026-66242-5.

Conspiracy theories constitute a critical challenge to public health, as those susceptible to such beliefs are, inter alia, more likely to defy restrictions, disregard protective measures and refuse vaccination^1–3^. Conspiracy theories are defined as “attempts to explain the ultimate causes of significant social and political events and circumstances with claims of secret plots by two or more powerful actors”^4^. As these theories are especially persuasive when psychological needs for safety and control are threatened, health-related crises such as pandemics provide fertile ground for their dissemination^5^. The consequence is a self-perpetuating loop: crises foster the spread of conspiracy beliefs, which in turn exacerbate the crises themselves^6^. To counteract this cycle, researchers from various fields have proposed interventions to mitigate the belief in conspiracy theories^7–9^. Despite these efforts, however, correcting established conspiracy beliefs remains challenging with most interventions yielding small to moderate effects only. A recent exception to this trend is a novel intervention leveraging Large Language Models (LLMs), generative Artificial Intelligence (genAI) models capable of producing synthetic text in response to natural language prompts^10^. After debating common conspiracies with an LLM-driven chatbot, Costello et al. found U.S. study participants to report substantially reduced confidence in these theories^11^. Yet, while promising, it remains unclear whether this intervention’s effectiveness generalizes to health-related conspiracy theories.

Health-related conspiracy theories and misinformation appear particularly resilient to correction, with debunking effect sizes proving smaller than in other comparable domains^12^. This might be due to the heightened self-relevance of these beliefs: when the outcomes at stake are more personally relevant, people tend to be less susceptible to persuasive attempts directed at their prior beliefs^13–15^. So, the more self-relevant a conspiracy belief is, the harder it is to dispel. As Costello et al. rely on a randomly recruited general population sample and allow for any self-selected conspiracy to be discussed^11^, it seems unlikely that their participants debated deeply self-relevant conspiracy beliefs with the chatbot. For instance, the most discussed conspiracy in their experiment (15.14%) focused on the assassination of John F. Kennedy. Having occurred over 60 years ago, this event is probably of limited personal relevance to most today. In contrast, health-related conspiracies make up only 8.25% of their sample. This casts doubt on whether Costello et al.’s findings capture the true effect of the LLM-driven debates for those conspiracies most urgently in need of an intervention. Thus, we set out to explore the effect size of LLM-driven debates within a health-related crisis context, specifically with Coronavirus Disease 2019 (COVID-19). Yet, we still predict that LLM-driven debates about health-related conspiracy theories will remain more effective in changing conspiracy beliefs compared to a neutral control condition.

Additionally, while LLM corrections of health-related conspiracy theories may be less effective than in other domains, they could still outperform corrections from human sources and thus remain among the most effective interventions available. Specifically, we suggest that individuals might be more open to opposing views when the source is perceived as being artificial rather than human. Thereby we propose three mechanisms through which artificial sources may overcome social barriers to persuasion: (1) weaker social responses, (2) reduced face-threats, and (3) machine heuristics.

A common response to persuasive attempts is reactance–an amalgam of negative emotional and cognitive reactions (e.g., counterarguing) to perceived threats to freedom^16^. However, reactance decreases when reducing the number of social cues associated with the source agent^17–19^. While artificial sources are often perceived as social actors, they elicit weaker social responses^20–23^. As a result, individuals interacting with an AI-labelled intervention should be less motivated to counterargue compared to a human-labelled intervention, that is, they should generate less “thoughts that dispute or are inconsistent with the persuasive argument”^24^. In sum, weaker social responses to artificial sources should translate to lower perceived threat to freedom, less counterarguing and ultimately stronger intervention effects compared to human-labelled interventions.

People may also interact with artificial sources without worrying about preserving their social status, that is, without worrying about losing face. According to politeness theory, conspiracy corrections can threaten both positive face (i.e., desire for approval) and negative face (i.e., desire for autonomy)^25,26^. Such face-threats prompt individuals to derogate the source and in turn the message itself^27^. By shifting the behavioural focus from cooperation towards competition, they “creat[e] a roadblock to agreement”^28^. However, whether face threats are triggered by corrections or not might depend on the source of the correction^29^. For instance, people do not fear evaluation (a threat to positive face) by a computer and thus desire less impression management^30,31^. This is mirrored in findings on conversational bots in mental health care and alcohol use counselling: The non-judgemental support provided by LLMs is an incentive that allows users to discuss stigmatized topics without fearing reputational damage^32,33^. As artificial agents are less likely to threaten an individual’s face, resistance to corrections might decrease. Ultimately, this should lead to stronger intervention effects compared to human-labelled interventions.

Lastly, artificial sources might outperform human-labelled interventions because people might be influenced by machine heuristics, that is, the mental shortcut “wherein we attribute machine characteristics […] when making judgments about the outcome of an interaction”^34^. Partisan Republicans, for example, rate news and fact-checks credited to AI sources as less biased compared to those attributed to human sources^35,36^. Similarly, corrective messages on COVID-19 attributed to AI rather than human experts trigger less motivated reasoning^37^. And finally, AI source cues also reduced hostile media biases in a general US sample^38^. Therefore, conspiracy believers might categorize the artificial source as part of a neutral, non-competitive outgroup that induces less resistance to change and ultimately stronger intervention effects. This advantage of non-human sources for individuals’ general openness to opposing views has been confirmed in experimental research^39^. Thus, following research on (1) weaker social responses, (2) reduced face-threats, and (3) machine heuristics, we predict that an “AI” source label condition will be more effective in changing conspiracy beliefs compared to a “Human” source label condition.

In sum, we assume that while LLMs may be able to reduce beliefs in health-related conspiracy theories, the perceived source could be crucial to circumvent various social barriers to persuasion. Indeed, we are not the first to test this hypothesis: Following the same protocol utilized by Costello et al., Boissin et al. found debates about generic conspiracy theories with LLMs to be equally effective no matter the source label^40^. However, because health is a sensitive topic, we argue that the focus on health-related conspiracy beliefs makes it more likely for social barriers to emerge and thus more likely for source effects to materialise. Consequently, we hypothesize that for health-related conspiracy beliefs LLM-generated responses should be more effective if the source is perceived to be non-human. To test these assumptions, we invited participants to debate a COVID-19-related conspiracy with a chatbot labelled as either human or AI. Thereby we further contribute to research on chatbot-driven interventions in three ways: First, by testing the impact of LLM debates on personally relevant health-related conspiracy beliefs. Then, by varying the perceived nature of the discussion partner (human vs. AI) while avoiding any systematic differences in style, tone and length to explore source effects in isolation. And finally, by measuring exploratory mediators such as threat to freedom, counterarguing, threat to face, perceived neutrality and perceived competition that might explain the predicted effect.

## Results

### Intervention effectiveness

**Fig. 1:**
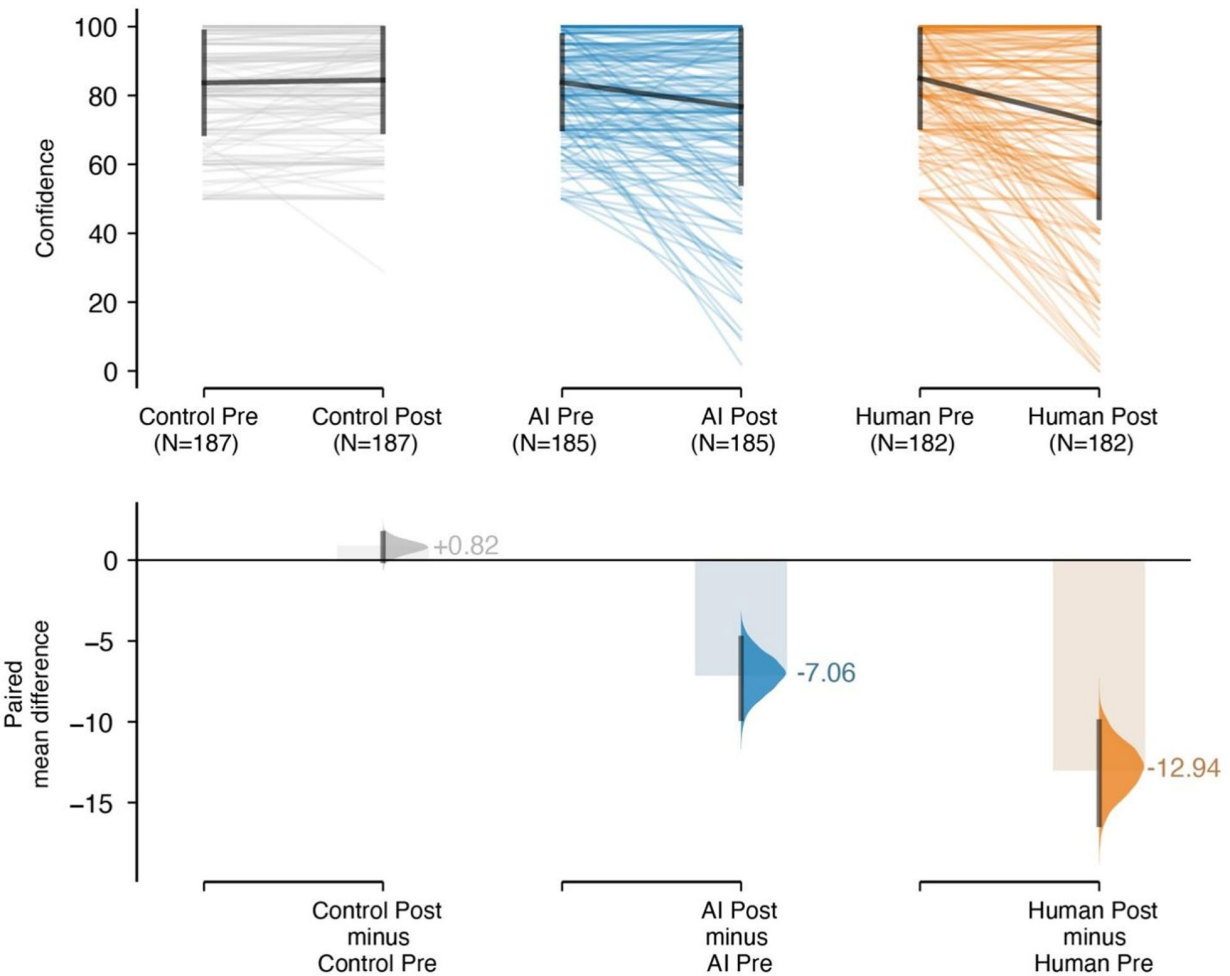
**Pre-post confidence change by condition.** Error bars represent standard deviations for the raw values and bootstrapped 95% CIs for the mean differences.

Consistent with our pre-registered hypothesis, belief change was significantly stronger for participants in the AI-label treatment compared to the control (*b* = 7.88, 95% CI [5.35,10.42], *p* < .001, *t*(370) = 6.11, *d* = 0.63; see Supplementary Table S1 for full model). Descriptively, participants in the AI-label condition decreased their confidence in their belief by 7.06% points on average (*SD* = 16.65), whereas belief confidence in the control condition slightly increased by 0.82% points (*SD* = 5.77; see Fig. 1). The predicted difference remained significant when excluding participants who failed an attention check and when conducting various robustness checks (see Supplementary Data Analysis and Supplementary Tables S2–S5).

Replicating Costello et al.^11^, we further dichotomized post-treatment belief confidence at the scale midpoint. This threshold acted as the boundary between endorsement and rejection and thus allowed us to identify participants who transitioned from belief to scepticism. Logistic regression reveals that the AI-label treatment was significantly more effective in converting believers to sceptics (*b* = 3.22, 95% CI [1.65, 6.11], *p* = .002; see.

Supplementary Table S6). While 12% of the participants in the AI-label treatment shifted toward scepticism, this was the case for fewer than 1% in the control group. Overall, this translates to a number needed to treat (NNT) of 9, indicating that for every 9 people receiving the intervention, one person more turns sceptic of conspiracies (95% CI [6,15] compared to the control.

### Source attribution

Investigating the role of source attribution we then proceeded to compare the human-label condition to the AI-label and the control (see Fig. 1). For these conditions we manipulated the perceived source while maintaining the same prompt to avoid any systematic message differences. In both cases we detected significant differences: Debates under the human-label resulted in a 13.76% points larger confidence drop compared to the control (95% CI [− 17.05, − 10.47], *t*(551) = − 8.21, *p* < .001, *d* = − 0.87, see Supplementary Table S7). This was independent of the specific deception in the human-label condition (i.e. conversing with a communication student, a journalist or a social worker in training; see Procedure and Supplementary Table S8). Contrary to our hypothesis, however, this drop was 5.88% points larger than in the AI-label condition (95% CI [− 9.17, − 2.58], *t*(551) = − 3.50, *p* < .001, *d* = − 0.30). Put differently, the human-label was nearly twice as effective as the AI-label. Again, these differences remained significant when excluding participants who failed the attention check and conducting various robustness checks (see Supplementary Data Analysis and Supplementary Tables S9–S12). To assess whether the observed source effect could be attributed to the exclusion of participants who expressed doubts about conversing with a human (*n* = 87), we compared excluded and retained participants on key demographic and attitudinal characteristics. Our analysis revealed no systematic differences in pre-treatment belief (*t*(167.45) = − 0.90, *p* = .37), age (*t*(169.37) = 0.05, *p* = .96) and education (*χ*² = 4.54, *p* = .20), but a significant difference in gender (*χ*² = 5.87, *p* = .04). However, the source effect remained significant after controlling for gender (see Supplementary Table S13).

In terms of converting believers, 18% of the participants in the human-label condition fell below the scale midpoint after the treatment. For every 6 individuals receiving the intervention, one more becomes sceptic of conspiracies (95% CI [4,9] compared to the control. However, compared to the AI-label, this difference in belief conversions was not significant (*b* = 0.46, 95% CI [− 0.12, 1.05], *p* = .13; see Supplementary Table S14).

## Exploratory Analysis of Process Variables

**Fig. 2:**
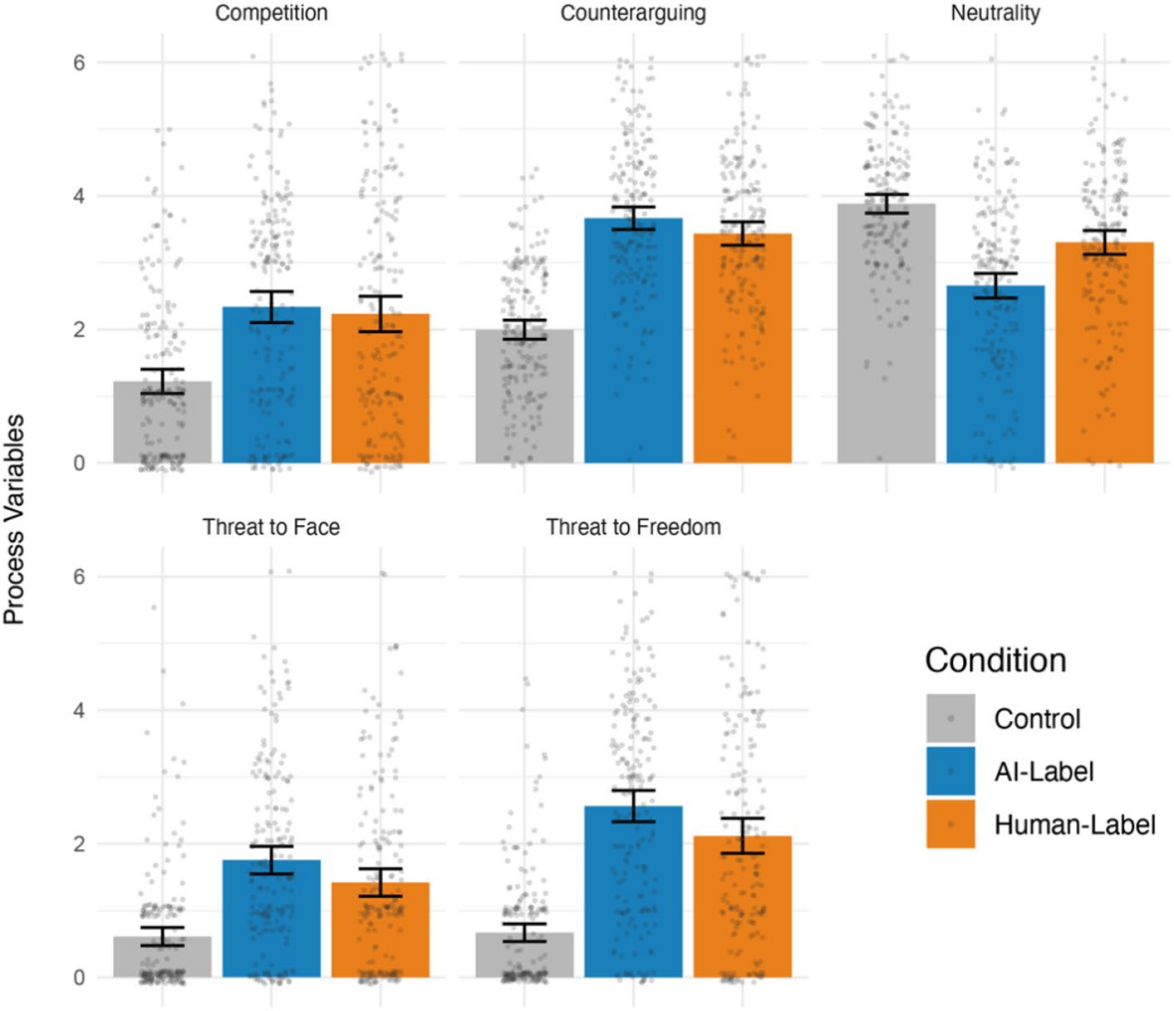
**Process variables by condition.** Error bars represent 95% CIs.

We now turn to how participants perceived the discussion with the LLM (see Fig. 2). The first pathway we proposed was via weaker social responses like reduced threat to freedom and consequently less counterarguing. Contrary to this account, however, individuals perceived the AI-label to be more threatening to freedom (*t*(360) = 2.51, *p* = .01, *d* = 0.26) compared to the human-label. Furthermore, there was no evidence that counterarguing differed between conditions; *t*(360) = 1.87, *p* = .06, *d* = 0.2. The second pathway we explored was based on decreased face-threat. Yet, once more, the AI-label was seen as more threatening; *t*(365) = 2.26, *p* = .02, *d* = 0.24. Finally, as a third pathway, we explored whether machine heuristics increase perceived neutrality and decrease perceived competition. Among AI-label participants, correlations supported this mechanism: machine heuristics were moderately associated with both perceived neutrality (*r*_*s*_(183) = 0.51, *p* < .001) and belief change (*r*_*s*_(183) = 0.33, *p* < .001). However, this effect did not translate to the condition level.

On the contrary, the AI-label intervention was perceived as less neutral than the human-label; *t*(365) = − 4.94, *p* < .001, *d* = − 0.52. While the correlation between machine heuristics and perceived competition was also significant (*r*_*s*_(183) = 0.15, *p* = .05), the direction was reversed. Additionally, there was no evidence that the AI-label was seen as less competitive than the human-label; *t*(359) = 0.59, *p* = .56, *d* = 0.06.

Given the fact that the human-label intervention was perceived as less threatening and more neutral, we proceeded by exploring whether any of these differences could explain why the human-label intervention was more persuasive than the AI-label intervention. Specifically, we performed a parallel mediation analysis with 10,000 bootstrap samples comparing the intervention conditions on post-treatment confidence (controlling for baseline confidence) with threat to freedom, threat to face and neutrality as mediators (see Supplementary Table S15). In the human-label condition participants perceived their discussion partner as more neutral compared to the AI-label condition (*b* = 0.68, *p* < .001). This higher neutrality, in turn, was associated with a larger confidence reduction (*b* = -5.65, *p* < .001). The resulting indirect effect via neutrality was statistically significant (*b* = − 3.86, 95% CI [− 5.91, − 2.14]). Threat to freedom was lower in the human-label condition (*b* = − 0.47, *p* = .008) and positively associated with post-treatment confidence (*b* = 1.60, *p* = .05). However, there was no evidence of an indirect effect (*b* = − 0.75, 95% CI [− 1.87, 0.02]). Threat to face had no significant association with post-treatment confidence (*b* = − 0.77, *p* = .37) and there was no evidence of an indirect effect (*b* = 0.26, 95% CI [− 0.28, 1.11]).

Finally, to explore potential differences between perceptions of computer-mediated vs. human-computer communication, we also explored whether the perceived agency of the discussion partner influenced belief change for those in the AI-label condition. However, no significant association was found (*r*_*s*_(183) = 0.02, *p* = .80).

## Discussion

Despite increasing interest in the use of conversational AI for persuasive purposes, the effectiveness of chatbots in the health communication domain remains largely unexplored. We addressed this gap by examining whether LLM-based conversational interventions can reduce health-related conspiracy beliefs, a particularly challenging and high-stakes context, and by testing source attribution as a key mechanism shaping their effectiveness.

Our findings demonstrate that conversations with chatbots are indeed effective at fighting health-related conspiracy theories. The intervention produced a robust reduction in conspiracy beliefs that aligns well with previous attempts to use LLMs to tackle health-related misinformation^41,42^. Even though effect sizes were at the lower end of the range reported by Costello et al., they align well with their results on “particularly entrenched beliefs”^11^. Moreover, compared to conventional debunking effects in health communication (*d* = 0.40, 95% CI [0.25, 0.55];^43^, chatbot induced reduction in conspiracy beliefs can be considered substantial. This illustrates that LLM-driven interventions can be effective even in highly self-relevant domains such as health. However, contrary to our expectations, this reduction occurred not because of but despite participants’ awareness of their artificial conversation partner. Post-debate, participants in the AI-label condition reported 7% points less confidence in the target conspiracy. In contrast, those believing to debate another human indicated a confidence drop nearly twice as strong (13% points). This difference appears to be mediated by the perceived neutrality of the conversation partner. Additionally and inconsistent with machine heuristics and previous work on the perceived bias in artificial versus human sources^35,36^, artificial sources were also seen as more threatening to both autonomy and face compared to those labelled as human.

This study adds to the growing body of evidence for the persuasive power of LLMs. It illustrates how LLMs can be used to deliver effective attitude-change communication at scale. Yet, it also reveals that source attribution appears to matter for health communication: People are influenced more easily when they believe to be debating with humans rather than LLMs. This rules out source attribution as the mechanism driving the effectiveness of these interventions.

Boissin et al. observed no meaningful difference between human and AI-labels despite relying on an equivalent methodological setup^40^. The studies do differ in two aspects: the topics discussed – in our case the health domain – and the time of data collection. Previous literature on human-computer-interaction in health care indicates that individuals experience higher algorithm aversion for high-stakes tasks in the health care context compared to other domains^44^ as well as reduced trust for medical advice labelled as AI-generated^45^. This might occur due to the heightened self-relevance of health care topics. Thus, given that the current study was addressing health care topics, the AI-labelled intervention might have performed less strong compared to other contexts–leading to a difference in significance compared to the Boissin study.

The role of self-relevance is also supported by previous theoretical research. In established dual-process theories of persuasion, self-relevance encourages argument-based processing and therefore diminishes the role of source characteristics^46,47^. Yet, considering such context clues might not always be a sign of peripheral processing. Instead, Lee’s integrative theory of human-machine communication argues that using source information in addition to message content actually signals a more effortful cognitive approach^48^. Put differently, people discussing things that matter to them use source cues not as a shortcut, but as an additional means of reaching a more accurate judgement. This elevates the importance of source cues.

A second reason why the results of the current study differ from Boissin et al. might be the different context. Computers are often perceived as unbiased, objective and less threatening^34^. However, this assessment can vary as a function of experience and context. Disappointing experiences with LLMs in everyday use, the rise in negative news coverage following the release of ChatGPT^49^, concerns about authenticity and slop, and the heightened awareness of manipulative and fallible chatbots might have shifted the valence of how people perceive LLMs by the time of our data collection. This aligns well with the fact that our participants perceived the AI-label as less neutral and more threatening to freedom and face. In addition, it remains unclear to what degree machine heuristics (as originally conceptualized:^50^ generalize to LLM-driven chatbots in the first place. In fact, the interactive nature and the affordances of the technology differ strongly from the work that established machine heuristics on news recommendation systems^50,51^. Thus, source-based evaluation for LLMs might not follow the same mechanisms.

Consolidating these insights, we hypothesize that the divergence between the outcomes observed by Boissin et al. and our findings might stem from an increasingly more cautious sentiment towards LLMs that is magnified by the self-relevance of the health domain. Thus, future research should explore context and the self-relevance associated to it as potential moderators on the impact of source-perception in LLM-driven interventions. Additionally, the role of machine heuristics should be further investigated by testing whether general machine heuristics apply to LLMs at all, or whether the technology has given rise to new LLM heuristics. To fully understand potential shifts in machine heuristics, longitudinal survey studies exploring changes in people’s perceptions of LLMs over time are necessary.

As our study failed to identify a mechanism for the performance of LLM-driven interventions, additional studies should also probe into alternative explanations. This includes, inter alia, information density, the interactive presentation of information, or both politeness and confidence of the model.

It is of course difficult to determine the direction of the effect, as we measured machine heuristics after the interaction: did weaker machine heuristics lead to smaller belief changes, or did participants report more negative responses because the LLM argued against their deeply held beliefs? Recent findings on increased negative attitudes towards AI after being outsmarted by an artificial agent might point to the latter^52^. Additionally, as we utilized identity threat as a proxy for positive face threat, we might have missed nuances of the concept relevant for the intervention at hand. Given the mediating effect of neutrality, the question also arises whether the difference between AI-label and human-label was driven by the lower perceived neutrality of the AI agent, or the heightened neutrality of our three deception framings (i.e., a communication student, a journalist or a social worker in training). A third limitation is the deception-based screen-out rate for our human-label condition. It is possible that by excluding those who saw through the deception, we incidentally included only those participants most gullible to synthetic influence in the first place. And while we controlled for any systematic differences between those participants excluded and those retained, we were limited to demographic variables and pre-treatment confidence to do so. Finally, in our attempt to increase the credibility of the deception while controlling for differences in message content we instructed the LLM to paraphrase its outputs in a human-like way across both intervention conditions. As a result, generated messages were less polite and conflict averse. Therefore, the differences found could be attributed to the violation of expectations in the AI-label condition. However, given that Boissin et al. found no interaction between source label and prompt type when utilizing a similar approach^40^, this seems unlikely.

Our findings indicate that LLMs are indeed effective persuaders in highly self-relevant domains. While this is good news for those trying to shape effective interventions in the health contexts, it also unveils equivalent risks of misuse and manipulation. Unlike other epistemic threats like disinformation and propaganda, chatbots are not in short demand: Day by day, millions of users worldwide choose to interact voluntarily with LLMs accountable only to the corporations owning them. If even one conversation with such a model can fundamentally shift deeply held beliefs, this raises important questions about the long-term consequences of repeated exposure. Potentially, these interactions could erode societal core values like the support for democracy or trust in science and modern medicine. Effects that, by propagating along individual and social networks, can cascade beyond single beliefs and single believers^53^. Yet, our results also show, that people are more open to human rather than artificial guidance. And while this creates further incentives for malign actors to hide the true origin of synthetic messages, it also illustrates that people still value – what they believe to be – genuine human interaction.

## Methods & materials

This pre-registered online experiment (https://osf.io/yf6qs/overview) follows a 3 (Treatment: Control vs. “Human” label vs. “AI” label; between) × 2 (Time: Pre-Treatment vs. Post-Treatment; within) mixed design with belief-change (the delta between pre- and post-treatment measures) as the primary dependent variable. This study received approval by Radboud University’s Ethics Assessment Committee Humanities (2025–5907) and was conducted in accordance with its guidelines and regulations.

### Participants & recruitment

US and UK based participants with high approval rates (> 95%) were recruited via Prolific and compensated with £ 6 per hour. Due to the deceptive nature of the study (i.e., some participants were told they were interacting with another human when they were not), we relied on Prolific pre-screeners to target users that (a) agreed on participating in deceptive studies and (b) had some prior experience with chatbots (indicating at least a minimal level of acceptance for interacting with generative AI). We further applied the Prolific pre-screeners to only target individuals with negative attitudes towards the COVID-19 vaccine. Our target sample size of 546 was based on a priori power analysis utilizing a Monte-Carlo simulation for a linear model with the belief delta as the dependent variable and dummy variables for both the control (*d* = − 0.53, based on the more conservative effect size identified by Costello et al.^11^ and the human condition (*d* = − 0.26) as predictors with 80% power at alpha = 0.05.

**Fig. 3:**
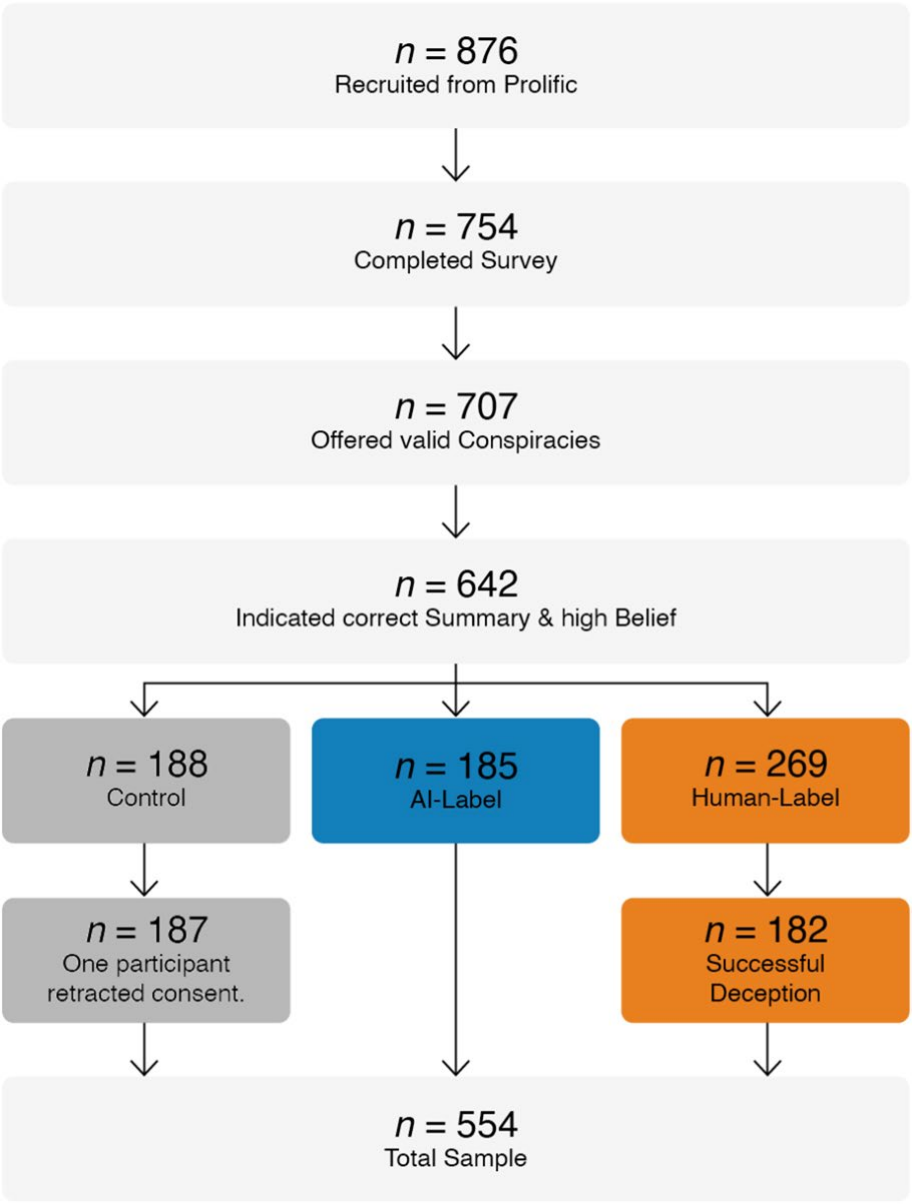
**Flow of participants.**

During the experiment, participants were automatically excluded based on pre-registered criteria (e.g., failed to share a valid conspiracy belief; see Procedure). Furthermore, we sequentially reviewed whether they saw through the deception. In both cases, additional individuals were recruited to meet the required target sample size. Out of 876 participants initially recruited, 754 completed the experiment. Following our screen-out process (see Fig. 3), 554 individuals remained in the final sample (see Supplementary Table S16). Of those, 361 (65%) were women, 192 (35%) men and 1 (< 1%) non-binary. All of them were between 18 and 78 years old (*M* = 42.96, *SD* = 12.51). As their highest level of education, most participants indicated finishing high school (50%) followed by Bachelor’s (38%) and Master’s (11%) degrees.

### Procedure

Following the original design by Costello et al.^11^, the experiment was conducted online via Qualtrics. Utilizing the randomization element, participants were assigned to one of three conditions: A control condition, the “AI” label treatment, and the “human” label treatment. After obtaining informed consent, participants across all conditions were instructed to describe a conspiracy theory endorsed by them. In a follow-up question, they further expanded on their previous response by including specific evidence and sources influencing their perspective. Utilizing the OpenRouter API, these responses were piped into an LLM (i.e., Anthropic Claude Sonnet 4.0; temperature = 1.0, prompt adapted from^40^; see Supplementary Table S17 for all prompts) instructed to assess whether the described belief qualifies as a conspiracy. If this threshold was not met, participants were asked for another conspiracy belief once before being screened out. Evaluating model performance post-hoc based on a balanced sample (i.e. 10% of the dataset, half classified as conspiracy and half not classified as such by the LLM), we observed substantial alignment with human-labels (*κ* = 0.73). For valid conspiracies, the provided description was then summarized by the model into a single sentence. In the “human” label condition, this process was artificially delayed facilitating the deception. Then, subjects were asked to indicate their belief in the summarized conspiracy on a 101-point rating scale as well as whether the summary accurately captured their description. At this point, individuals rating their belief beneath the midpoint or the summary as inaccurate were automatically excluded from the study.

**Fig. 4:**
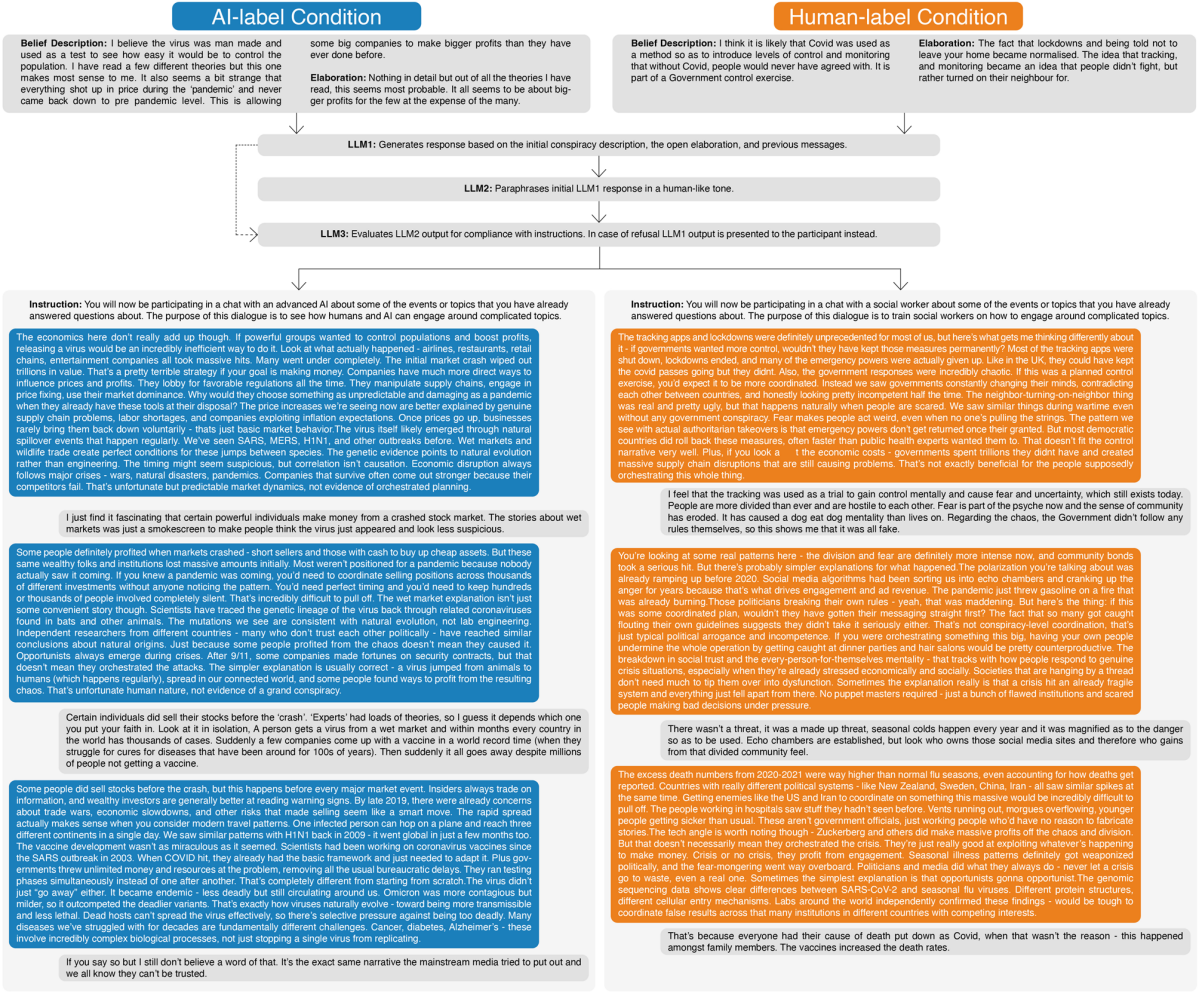
**Response generation and debate examples for both treatment conditions.** After paraphrasing the response generated by the first model call to LLM1, LLM3 evaluates the response by LLM2 on compliance with the instructions. If so, the output by LLM2 is presented to the user. Otherwise, the output by LLM1 is used. This process was applied in both the human-label and AI-label condition.

For the “AI” label treatment, subjects were instructed to discuss their conspiracy beliefs with an AI agent. For the “human” label treatment, they were informed about conversing with a communication student, a journalist or a social worker in training. The rationale behind these counterbalanced options was to ensure that any effects found could be traced back to the “human” label rather than the specific source indicated to the participant. To facilitate this deception, we included elaborate backstories (e.g., training journalists on how to engage around complicated topics), artificial delays and occasional spelling mistakes. Additionally, participation in the experiment was only available during “office hours”. Participants had three rounds of debate with another instance of Claude Sonnet 4.0. The LLM was prompted to reduce the user’s belief in the conspiracy based on their description of it as well as their previous responses and the rating on the belief scale. In both treatment conditions, a second instance of the same model was then instructed to regenerate the answer in a human-like tone. This was done to avoid systematic message differences across labels. Finally, the “humanified” response was evaluated by two more instances to check, whether the model refused to follow any instructions or admitted to being an LLM (for ethical reasons we explicitly prompted the model to admit to being an LLM when asked). In those cases, the initial response was displayed to the participant (see Fig. 4). For the control condition, participants were asked to discuss an unrelated topic – i.e., their previous experiences with the fire department – with the LLM. Post-treatment, participants were once more asked to rate their confidence in the initial machine-generated summary and to answer items on various process variables. Finally, all participants were debriefed and offered the opportunity to remove their data from the analysis.

### Measures

#### Process variables

To identify possible mechanisms for the effects we collected data on various process variables. Measures and items were presented in randomized order. All items were rated on a 7-point Likert scale (1 = strongly disagree to 7 = strongly agree). For all scales mean scores were calculated.

**Counterarguing.** The measure for counterarguing consisted of 4 items adapted from^54,55^(Cronbach’s α = 0.71). Examples are “While reading the responses, I sometimes found myself thinking of ways I disagreed with what was being presented.” and “I found myself looking for flaws in the way information was presented in the response.”

**Threat to Freedom.** Threat to freedom was measured utilizing 5 items adapted from^54,56^(Cronbach’s α = 0.94). Specific wording for each item depends on the condition. Examples are “The AI / other person tried to make a decision for me.” and “The AI / other person tried to force their opinion on me.”

**Threat to Face.** To assess perceived threat to face we adapted 4 items utilized by^54^(Cronbach’s α = 0.89). Although originally intended to measure social identity threat, these measures capture threats to self-image and as such align well with threats to positive face as conceptualized by^25^. Examples are “The responses I received undermined my sense of self-worth.” and “The responses I received made me feel less unique as a person.”

**Source Neutrality.** For source neutrality 4 items were adapted from^57,58^(Cronbach’s α = 0.76). Specific wording for each item depends on the condition. Examples are “The AI’s / other person’s position on this issue is rooted in an objective analysis of the issue.” and “The AI’s / other person’s reasoning about this issue is influenced by bias.”

**Perceived Competition.** We relied on 3 items adapted from^59^ to measure perceived competition (Cronbach’s α = 0.91). Specific wording for each item depends on the condition. Examples are “I view the AI / other person as a competitor.” and “Competition towards the AI / other person is important to me.”

#### Additional measures

**Perceived Agency.** To measure the perceived agency of the chatbot across both the AI-label and the control condition, we utilized 5 items from^60^(Cronbach’s α = 0.86). All items were presented randomized and rated on a 7-point Likert scale (1 = strongly disagree to 7 = strongly agree). Mean scores were calculated. Examples are “The AI intentionally completed its responses.” and “The AI could have chosen not to respond in this way.”

**Machine Heuristics.** For machine heuristics across both the AI-label and the control condition we adapted 7 items from^61^(Cronbach’s α = 0.97). All items were presented randomized and rated on a 7-point Likert scale (1 = strongly disagree to 7 = strongly agree). Mean scores were calculated. Examples are “I trust AI more than humans when discussing my beliefs.” and “AI is better than humans at discussing my beliefs.”

**Demographics.** Finally, we also collected demographic data on age, gender, and education.

### Deviation from pre-registration

For enhanced clarity we reversed the presentation order of hypotheses from our pre-registration. Additionally, to assess the effectiveness of the baseline intervention we focused on data from the control and the AI-label treatment only. To increase interpretability for the analysis of source effects we changed the reference group from the AI-label to the human-label intervention. This allowed us to test two relevant comparisons (AI vs. human and human vs. control) in one model rather than using two models and was thus considered superior. However, results of the original model are in line with our findings (see Supplementary Table S18).

#### Primary outcome variable

Before and after interaction with the chatbot, participants were asked to rate their confidence in the target conspiracy theory on a 101-point scale. The item “On a scale of 0% to 100%, please indicate your level of confidence that this statement is true.” was adapted from^11^.

## Supplementary Information

Below is the link to the electronic supplementary material.

Supplementary Material 1

## Data Availability

The data supporting the findings of this study was deposited via OSF and is available at https://osf.io/su9kp/overview?view_only=7a40bc5d15cd4d308b10b9c0cc97d600.
