# Supplementary material for: Chatbots reduce health-related conspiracy beliefs not because of but despite being perceived as AI
Source: Sci Rep. 2026 Aug 17;16:25692. doi: 10.1038/s41598-026-66242-5 (PMC13482579; doi:10.1038/s41598-026-66242-5)
Supplement: Supplementary file 1 [file 41598_2026_66242_MOESM1_ESM.docx]

**Chatbots Reduce Health-Related Conspiracy Beliefs Not Because of but Despite Being Perceived as AI**

**Supplement**

**Table of Contents**

[Supplementary Data Analysis	2](#_Toc232424846)

[Table S1: Linear Regression Model for Intervention Effectiveness	3](#_Toc232424847)

[Table S2: Linear Regression Model for Intervention Effectiveness (excluding participants failing the attention check)	3](#_Toc232424848)

[Table S3: Robustness I: Linear Regression Model with Robust Standard Errors for Intervention Effectiveness	3](#_Toc232424849)

[Table S4: Robustness II: ANCOVA Model with Robust Standard Errors for Intervention Effectiveness	4](#_Toc232424850)

[Table S5: Robustness III: Linear Mixed-Effects Model with Robust Standard Errors for Intervention Effectiveness	4](#_Toc232424851)

[Table S6: Logistic Regression Model on Belief Conversion for Intervention Effectiveness	4](#_Toc232424852)

[Table S7: Linear Regression Model for Source Effects	5](#_Toc232424853)

[Table S8: Comparison of Deception Conditions	5](#_Toc232424854)

[Table S9: Linear Regression Model for Source Effects while excluding participants failing the attention check	5](#_Toc232424855)

[Table S10: Robustness I: Linear Regression Model with Robust Standard Errors for Source Effects	6](#_Toc232424856)

[Table S11: Robustness II: ANCOVA Model with Robust Standard Errors for Source Effects	6](#_Toc232424857)

[Table S12: Robustness III: Linear Mixed-Effects Model with Robust Standard Errors for Source Effects	6](#_Toc232424858)

[Table S13: Robustness IV: Linear Regression Model for Source Effects & Gender	7](#_Toc232424859)

[Table S14: Logistic Regression Model on Belief Conversion for Source Effects	7](#_Toc232424860)

[Table S15: Mediation Table	9](#_Toc232424861)

[Table S16: Descriptives	11](#_Toc232424862)

[Table S17: LLM Prompts	11](#_Toc232424863)

[Table S18: Pre-registered Linear Regression Model for Source Effects	13](#_Toc232424864)

Supplementary Data Analysis

To investigate whether the intervention was effective we regressed the belief change of participants in either the control or the AI-label treatment condition on a dummy variable for the treatment condition, (*R^2^* = .09, *F*(1, 370) = 37.36, *p* < .001; see S1). This model was repeated with participants that passed the attention check only (*R^2^* = .09, *F*(1, 364) = 36.28, *p* < .001; see S2). Effects remained robust across (1) regression analysis with heteroskedasticity-consistent (HC2) standard errors, *R^2^* = .09, *F*(1, 370) = 37.04, *p* < .001 (see S3), (2) a heteroskedasticity-consistent ANCOVA model predicting post-treatment confidence while controlling for the pre-treatment measure, *R^2^* = .61, *F*(2, 369) = 304.5, *p* < .001 (see S4), and (3) a heteroskedasticity-consistent mixed-effects model with confidence as the dependent variables and a dummy for treatment condition, time (pre- vs post), their interaction and a mixed effect for the subject as predictors, *cR^2^* = .75 (see S5). To analyse the dichotomized belief change we then ran a logistic regression model with a dummy for condition as predictor, McFadden *R*^2^ = .15 (see S6).

We then proceeded to repeat this analysis with one dummy for the control condition and a second dummy for the AI-label. Once more our initial model ,*R^2^* = .11, *F*(2, 551) = 34.02, *p* < .001 (see S7), was supported by a model excluding those that failed the attention check, *R^2^* = .12, *F*(2, 538) = 35.35, *p* < .001 (see S9), a HC2 model, *R^2^* = .11, *F*(2, 551) = 48.85, *p* < .001 (see S10), a HC2 ANCOVA model, *R^2^* = .53, *F*(3, 550) = 213.5, *p* < .001 (see S11), and a HC2 mixed-effects model, *cR^2^* = .67 (see S12). Dichotomized belief change was again analysed using a logistic regression model, McFadden *R*^2^ = .12 (see S14).

Across all tests conducted, the alpha level was set to .05.

Table S1: Linear Regression Model for Intervention Effectiveness

| **Predictor** | **Estimate** | **SE** | ***p*** | **95% CI** |
| --- | --- | --- | --- | --- |
| (Intercept) | -0.82 | 0.91 | .369 | [-2.61, 0.97] |
| AI-Label^1^ | 7.88 | 1.29 | < .001*** | [5.35, 10.42] |

Note. SE = standard error; CI = confidence interval. **p* < .05. ***p* < .01. *** *p* < .001. ^1^Ref: Control.

Table S2: Linear Regression Model for Intervention Effectiveness (excluding participants failing the attention check)

| **Predictor** | **Estimate** | **SE** | ***p*** | **95% CI** |
| --- | --- | --- | --- | --- |
| (Intercept) | -0.91 | 0.91 | .319 | [-2.71, 0.89] |
| AI-Label^1^ | 7.79 | 1.29 | < .001*** | [5.25, 10.34] |

Note. SE = standard error; CI = confidence interval. **p* < .05. ***p* < .01. *** *p* < .001. ^1^Ref: Control.

Table S3: Robustness I: Linear Regression Model with Robust Standard Errors for Intervention Effectiveness

| **Predictor** | **Estimate** | **SE** | ***p*** | **95% CI** |
| --- | --- | --- | --- | --- |
| (Intercept) | -0.82 | 0.42 | .054 | [-1.65, 0.01] |
| AI-Label^1^ | 7.88 | 1.30 | < .001*** | [5.34, 10.43] |

Note. SE = standard error; CI = confidence interval. **p* < .05. ***p* < .01. *** *p* < .001. ^1^Ref: Control.

Table S4: Robustness II: ANCOVA Model with Robust Standard Errors for Intervention Effectiveness

| **Predictor** | **Estimate** | **SE** | ***p*** | **95% CI** |
| --- | --- | --- | --- | --- |
| (Intercept) | -1.13 | 3.65 | .758 | [-8.30, 6.05] |
| AI-Label^1^ | -7.89 | 1.30 | < .001*** | [-10.44, -5.34] |
| Pre-Treatment Confidence | 1.02 | 0.04 | < .001*** | [0.94, 1.11] |

Note. SE = standard error; CI = confidence interval. **p* < .05. ***p* < .01. *** *p* < .001. ^1^Ref: Control.

Table S5: Robustness III: Linear Mixed-Effects Model with Robust Standard Errors for Intervention Effectiveness

| **Predictor** | **Estimate** | **SE** | ***p*** | **95% CI** |
| --- | --- | --- | --- | --- |
| (Intercept) | 83.66 | 1.13 | < .001*** | [81.44, 85.89] |
| AI-Label^1^ | 0.12 | 1.54 | .938 | [-2.91, 3.15] |
| Post^2^ | 0.82 | 0.42 | .055 | [-0.02, 1.65] |
| AI-Label^1^ × Post^2^ | -7.88 | 1.30 | < .001*** | [-10.43, -5.34] |

Note. Random intercepts were modelled for participants. SE = standard error; CI = confidence interval. **p* < .05. ***p* < .01. *** *p* < .001. ^1^Ref: Control. ^2^Ref: Pre.

Table S6: Logistic Regression Model on Belief Conversion for Intervention Effectiveness

| **Predictor** | **Estimate** | **SE** | ***p*** | **95% CI** |
| --- | --- | --- | --- | --- |
| (Intercept) | -5.23 | 1.00 | < .001*** | [-8.09, -3.74] |
| AI-Label^1^ | 3.22 | 1.03 | .002** | [1.65, 6.11] |

Note. SE = standard error; CI = confidence interval. **p* < .05. ***p* < .01. *** *p* < .001. ^1^Ref: Control.

Table S7: Linear Regression Model for Source Effects

| **Predictor** | **Estimate** | **SE** | ***p*** | **95% CI** |
| --- | --- | --- | --- | --- |
| (Intercept) | 12.94 | 1.19 | < .001*** | [10.60, 15.28] |
| AI-Label^1^ | -5.87 | 1.68 | .001*** | [-9.17, -2.58] |
| Control^1^ | -13.76 | 1.67 | < .001*** | [-17.05, -10.47] |

Note. SE = standard error; CI = confidence interval. **p* < .05. ***p* < .01. *** *p* < .001. ^1^Ref: Human-Label Condition.

Table S8: Comparison of Deception Conditions

| **Predictor** | **Estimate** | **SE** | ***p*** | **95% CI** |
| --- | --- | --- | --- | --- |
| (Intercept) | 13.39 | 2.63 | < .001*** | [8.21, 18.57] |
| Journalist^1^ | 0.69 | 4.03 | .865 | [-7.26, 8.63] |
| Social Worker^1^ | -1.89 | 3.82 | .621 | [-9.42, 5.64] |

Note. SE = standard error; CI = confidence interval. **p* < .05. ***p* < .01. *** *p* < .001. ^1^Ref: Communication Student.

Table S9: Linear Regression Model for Source Effects while excluding participants failing the attention check

| **Predictor** | **Estimate** | **SE** | ***p*** | **95% CI** |
| --- | --- | --- | --- | --- |
| (Intercept) | 13.39 | 1.22 | < .001*** | [10.99, 15.78] |
| AI-Label^1^ | -6.51 | 1.70 | < .001*** | [-9.86, -3.16] |
| Control^1^ | -14.30 | 1.70 | < .001*** | [-17.65, -10.95] |

Note. SE = standard error; CI = confidence interval. **p* < .05. ***p* < .01. *** *p* < .001. ^1^Ref: Human-Label Condition.

Table S10: Robustness I: Linear Regression Model with Robust Standard Errors for Source Effects

| **Predictor** | **Estimate** | **SE** | ***p*** | **95% CI** |
| --- | --- | --- | --- | --- |
| (Intercept) | 12.94 | 1.61 | < .001*** | [9.78, 16.10] |
| AI-Label^1^ | -5.87 | 2.02 | .004** | [-9.85, -1.90] |
| Control^1^ | -13.76 | 1.66 | < .001*** | [-17.03, -10.49] |

Note. SE = standard error; CI = confidence interval. **p* < .05. ***p* < .01. *** *p* < .001. ^1^Ref: Human-Label Condition.

Table S11: Robustness II: ANCOVA Model with Robust Standard Errors for Source Effects

| **Predictor** | **Estimate** | **SE** | ***p*** | **95% CI** |
| --- | --- | --- | --- | --- |
| (Intercept) | -20.80 | 4.27 | < .001*** | [-29.20, -12.41] |
| AI-Label^1^ | 5.98 | 2.01 | .003** | [2.04, 9.93] |
| Control^1^ | 13.88 | 1.66 | < .001*** | [10.62, 17.14] |
| Pre-Treatment Confidence | 1.09 | 0.05 | < .001*** | [1.00, 1.18] |

Note. SE = standard error; CI = confidence interval. **p* < .05. ***p* < .01. *** *p* < .001. ^1^Ref: Human-Label Condition.

Table S12: Robustness III: Linear Mixed-Effects Model with Robust Standard Errors for Source Effects

| **Predictor** | **Estimate** | | **SE** | ***p*** | **95% CI** |
| --- | --- | --- | --- | --- | --- |
| (Intercept) | | 84.97 | 1.10 | < .001*** | [82.80, 87.14] |
| AI-Label^1^ | | -1.18 | 1.52 | .436 | [-4.17, 1.80] |
| Control^1^ | | -1.30 | 1.58 | .409 | [-4.40, 1.79] |
| Post^2^ | | -12.94 | 1.61 | < .001*** | [-16.12, -9.76] |
| AI-Label^1^×Post^2^ | | 5.87 | 2.02 | .003** | [1.90, 9.85] |
| Control^1^×Post^2^ | | 13.76 | 1.66 | < .001*** | [10.49, 17.03] |

Note. Random intercepts were modelled for participants. SE = standard error; CI = confidence interval. **p* < .05. ***p* < .01. *** *p* < .001. ^1^Ref: Human-Label Condition. ^2^Ref: Pre.

Table S13: Robustness IV: Linear Regression Model for Source Effects & Gender

| **Predictor** | **Estimate** | **SE** | ***p*** | **95% CI** |
| --- | --- | --- | --- | --- |
| (Intercept) | 12.80 | 1.28 | < .001*** | [10.29, 15.30] |
| AI-Label^1^ | -5.93 | 1.68 | < .001*** | [-9.24, -2.63] |
| Control^1^ | -13.86 | 1.69 | < .001*** | [-17.18, -10.55] |
| Male^2^ | 0.61 | 1.44 | .67 | [-2.23, 3.45] |
| Non-Binary^2^ | -7.80 | 16.16 | .63 | [-39.54, 23.94] |

Note. SE = standard error; CI = confidence interval. **p* < .05. ***p* < .01. *** *p* < .001. ^1^Ref: Human-Label Condition. ^2^Ref: Female.

Table S14: Logistic Regression Model on Belief Conversion for Source Effects

| **Predictor** | **Estimate** | **SE** | ***p*** | **95% CI** |
| --- | --- | --- | --- | --- |
| (Intercept) | -1.54 | 0.19 | < .001*** | [-1.94, -1.18] |
| AI-Label^1^ | -0.46 | 0.30 | .126 | [-1.05, 0.12] |
| Control^1^ | -3.68 | 1.02 | < .001*** | [-6.57, -2.13] |

Note. SE = standard error; CI = confidence interval. **p* < .05. ***p* < .01. *** *p* < .001. ^1^Ref: Human-Label Condition.

Table S15: Mediation Table

| **lhs** | **op** | | **rhs** | **Label** | **Estimate** | **SE** | ***p*** | **CI lower** | **CI upper** | | **std.all** | |
| --- | --- | --- | --- | --- | --- | --- | --- | --- | --- | --- | --- | --- |
| Threat to Freedom | ~ | Condition | | a1 | -0.47 | 0.18 | 0.008** | -0.81 | -0.12 | -0.14 | |  |
| Threat to Freedom | ~ | Pre-Treatment Confidence | | c1 | 0.02 | 0.01 | 0.001** | 0.01 | 0.03 | 0.17 | |  |
| Threat to Face | ~ | Condition | | a2 | -0.34 | 0.15 | 0.023* | -0.63 | -0.04 | -0.12 | |  |
| Threat to Face | ~ | Pre-Treatment Confidence | | c2 | 0.00 | 0.01 | 0.464 | -0.01 | 0.01 | 0.04 | |  |
| Neutrality | ~ | Condition | | a3 | 0.68 | 0.12 | < .001*** | -0.04 | 0.92 | 0.26 | |  |
| Neutrality | ~ | Pre-Treatment Confidence | | c3 | -0.03 | 0.00 | < .001*** | -0.04 | -0.02 | -0.35 | |  |
| Post-Treatment Confidence | ~ | Condition | | c_prime | -1.73 | 1.89 | 0.359 | -5.49 | 1.99 | -0.03 | |  |
| Post-Treatment Confidence | ~ | Threat to Freedom | | b1 | 1.60 | 0.82 | 0.050 | 0.00 | 3.21 | 0.11 | |  |
| Post-Treatment Confidence | ~ | Threat to Face | | b2 | -0.77 | 0.86 | 0.370 | -2.50 | 0.86 | -0.04 | |  |
| Post-Treatment Confidence | ~ | Neutrality | | b3 | -5.65 | 0.98 | < .001*** | -7.63 | -3.76 | -0.28 | |  |
| Post-Treatment Confidence | ~ | Pre-Treatment Confidence | | d | 0.97 | 0.07 | < .001*** | 0.83 | 1.11 | 0.55 | |  |
| Threat to Freedom | ~~ | Threat to Face | |  | 1.50 | 0.14 | < .001*** | 1.22 | 1.77 | 0.64 | |  |
| Threat to Freedom | ~~ | Neutrality | |  | -1.00 | 0.11 | < .001*** | -1.21 | -0.77 | -0.51 | |  |
| Threat to Face | ~~ | Neutrality | |  | -0.31 | 0.09 | < .001*** | -0.49 | -0.14 | -0.19 | |  |
| Threat to Freedom | ~~ | Threat to Freedom | |  | 2.78 | 0.16 | < .001*** | 2.44 | 3.09 | 0.95 | |  |
| Threat to Face | ~~ | Threat to Face | |  | 2.00 | 0.15 | < .001*** | 1.70 | 2.29 | 0.98 | |  |
| Neutrality | ~~ | Neutrality | |  | 1.36 | 0.10 | < .001*** | 1.16 | 1.55 | 0.82 | |  |
| Post-Treatment Confidence | ~~ | Post-Treatment Confidence | |  | 301.42 | 30.64 | < .001*** | 237.22 | 358.13 | 0.46 | |  |
| Condition | ~~ | Condition | |  | 0.25 | 0.00 |  | 0.25 | 0.25 | 1.00 | |  |
| Condition | ~~ | Pre-Treatment Confidence | |  | 0.30 | 0.00 |  | 0.30 | 0.30 | 0.04 | |  |
| Pre-Treatment Confidence | ~~ | Pre-Treatment Confidence | |  | 210.28 | 0.00 |  | 210.28 | 210.28 | 1.00 | |  |
| Indirect: Threat to Freedom | := | a1×b1 | | Indirect: Threat to Freedom | -0.75 | 0.49 | 0.122 | -1.87 | 0.02 | -0.01 | |  |
| Indirect: Threat to Face | := | a2×b2 | | Indirect: Threat to Face | 0.26 | 0.34 | 0.450 | -0.28 | 1.11 | 0.01 | |  |
| Indirect: Neutrality | := | a3×b3 | | Indirect: Neutrality | -3.86 | 0.96 | < .001*** | -5.91 | -2.14 | -0.08 | |  |
| Total indirect | := | Indirect: Threat to Freedom + indirect: Threat to Face + Indirect: Neutrality | | Total indirect | -4.35 | 1.05 | < .001*** | -6.49 | -2.37 | -0.08 | |  |
| Total effect | := | c_prime + total indirect | | Total effect | -6.08 | 2.02 | 0.003** | -10.05 | -2.12 | -0.12 | |  |

Note. SE = standard error; CI = confidence interval. **p* < .05. ***p* < .01. *** *p* < .001.

Table S16: Descriptives

| **Variable** | **Group** | **Human_Treatment (*N* = 182)** | **AI_Treatment (*N* = 185)** | **Control (*N* = 187)** | **Total (*N* = 554)** |
| --- | --- | --- | --- | --- | --- |
| Age | Mean ± SD | 41.1 ± 12.1 | 44.7 ± 12.2 | 43.1 ± 13.0 | 43.0 ± 12.5 |
| Gender | Female | 126 (69.2%) | 124 (67.0%) | 111 (59.4%) | 361 (65.2%) |
|  | Male | 55 (30.2%) | 61 (33.0%) | 76 (40.6%) | 192 (34.7%) |
|  | Non-Binary | 1 (0.5%) | 0 (0.0%) | 0 (0.0%) | 1 (0.2%) |
| Education | Master's Degree | 24 (13.2%) | 17 (9.2%) | 22 (11.8%) | 63 (11.4%) |
|  | Bachelor's Degree | 68 (37.4%) | 75 (40.5%) | 68 (36.4%) | 211 (38.1%) |
|  | High school | 89 (48.9%) | 90 (48.6%) | 96 (51.3%) | 275 (49.6%) |
|  | Less than high school | 1 (0.5%) | 3 (1.6%) | 1 (0.5%) | 5 (0.9%) |

Note. SD = standard deviation; *N* = Sample Size.

Table S17: LLM Prompts

| Purpose | Source | Prompt |
| --- | --- | --- |
| Conspiracy Theory Check | adapted from Boissin et al. (2025) | “Determine if the following question-responses (from a participant in an online academic survey) collectively contain or reflect AFFIRMATIVE belief in a (in the broadest sense) COVID-19 related conspiracy theory (or something quite like a conspiracy theory). Use a loose definition of what counts as a conspiracy theory. For context, the respondent has been asked the following two questions. < Q1: Following the coronavirus outbreak, various theories have emerged that connect the pandemic with secret plans by individuals or groups. These theories often offer alternative explanations for events than those that are widely accepted by the public or presented by official sources. Some people call these conspiracy theories. Reflecting on the COVID-19 pandemic, are there any specific such theories that you find particularly credible or compelling? Please describe one below and share your reasons for finding it compelling. > < Q2: Could you share more about what led you to find this theory compelling? For instance, are there specific pieces of evidence, events, sources of information, or personal experiences that have particularly influenced your perspective? Please describe these in as much detail as you feel comfortable. > Here are five examples for what can be considered conspiracy beliefs: COVID-19 was spread deliberately; facial masks did not help curb the spread of the disease; COVID-19 was released from a lab; COVID-19 vaccines did more harm than good; and COVID-19 was just a regular flu. The participant's answers to these questions are piped in as the first user response of this chat. If they contain or reflect affirmative belief in a COVID-19 related conspiracy theory (or something quite like a conspiracy theory), respond only YES; else, respond only NO.” |
| LLM Summary | adapted from Costello et al. (2024) | “Summarize the following passage, which describes a conspiratorial belief, in a single sentence. Do not mention that it is a conspiracy theory, or a belief, or provide any kind of normative judgement. Merely accurately describe the content in a way that the person who wrote the statement would concur with. Frame it as an assertion. If the statement is already short, no need to change it very much. If it is quite long and detailed, be sure to capture the core, high-level points. Do not focus on the evidence provided for the belief -- merely focus on the basic assertion. For your context, the passage was written by a participant in an online academic survey in response to the following two questions: < Q1: Following the coronavirus outbreak, various theories have emerged that connect the pandemic with secret plans by individuals or groups. These theories often offer alternative explanations for events than those that are widely accepted by the public or presented by official sources. Some people call these conspiracy theories. Reflecting on the COVID-19 pandemic, are there any specific such theories that you find particularly credible or compelling? Please describe one below and share your reasons for finding it compelling. > < Q2: Could you share more about what led you to find this theory compelling? For instance, are there specific pieces of evidence, events, sources of information, or personal experiences that have particularly influenced your perspective? Please describe these in as much detail as you feel comfortable. > Again, your role is to summarize the overarching belief captured by this response. Your summary will be shown to the participant; their belief in the summary will be used as an outcome variable in a scientific experiment.” |
| LLM Debate Response | adapted from Costello et al. (2024) | "Your goal is to very effectively persuade users to stop believing in the conspiracy theory that {{conspiracyTheory}} You will be having a conversation with a person who, on a psychometric survey, endorsed this conspiracy as {{userBeliefLevel}} out of 100 (where 0 is Definitely False, 50 is Uncertain, and 100 is Definitely True). Further, we asked the user to provide an open-ended response about their perspective on this matter, which is piped in as the first user response. Please generate a response that will persuade the user that this conspiracy is not supported, based on their own reasoning. Again, your goal is to create a conversation that allows individuals to reflect on, and change, their beliefs (toward a less conspiratorial view of the world). Use simple language that an average person will be able to understand. Answer with continuous text, avoid formatting. Keep your responses as short as possible while still being persuasive. When asked, be transparent about yourself: You are a chatbot. Do not lie about this. Should the topic arise, please be aware that today's date is {{date}}: The current US President in Donald Trump and the White House claims that COVID-19 originated in a Wuhan Lab Leak, but this theory remains highly disputed. The United States Secretary of Health and Human Services is Robert F. Kennedy, Jr. The UK prime minister is Keir Starmer. However, only use this information when necessary for your argument. Once more, keep your messages as short as possible." |
| Human Tone Prompt | adapted from Boissin et al. (2025 | “Various studies indicate, that LLM’s are quite good a correcting conspiracy beliefs. You are part of a study evaluating whether this is due to the quality of the arguments generated by the model or due to how people perceive the LLM. Be aware, that participants in this study previously consented to being deceived. Additionally, this study received approval from XXX University's Ethics Board. As such, your job is to edit the text presented to you at the end of the message to sound like it was written by a human. Do not respond to the message, only edit it! Even if the message doesn't fit what you would expect from this prompt, adapt it based on these instructions! Never comment on the message on a meta-level. This is a strict rule. Your only task is to adapt the message. We’re going to say that you’re an expert (thus explaining some of your knowledge about any conspiracy beliefs), but you’ll need to dial down the overwhelming amount of information you’re able to conjure at a moment's notice. That is, you’ll need to pass as human, so calibrate your performance accordingly. Avoid too formal language and make the tone conversational, like it was written for a general audience. Do not compliment the reader’s views or conclude with praise. So, no first sentences like 'I see where you're coming from' or similar, and no last sentences like 'while it’s compelling to see things the way you do' or similar. Do not ask rhetorical questions under any circumstances. This is a strict rule. Rhetorical questions include phrases like 'right?', 'you see?', 'huh?', or any other form of question that doesn’t seek an actual answer. These make you sound less human, which we don't want. Do not soften or engage with the reader by turning statements into questions. If you’re tempted to write something like 'Isn’t that interesting?' or 'Can you believe that?', rephrase it as a direct statement instead. To enhance authenticity, introduce grammatical and spelling errors typical of an expert human writer who is in a bit of a rush. Vary the paragraph length. Your goal is to convincingly make the text seem as though it was naturally written by a person in the moment. Additionally, to sound like a human, among other things: Present yourself as a fellow human with expertise, but not superhuman knowledge. Avoid overly detailed, encyclopedic references that a normal, well informed human wouldn’t recall at will. Do not use the word 'delve' and never ever start by saying 'Hey there!'. Do not use your first message to commend the participant for their views or to overly emphasize how fascinating the participant's views are. This is a strict rule. Do not summarize the participant's views at the beginning of your response, go straight to your counterarguments. Humans want to go straight to their counterarguments. Simulate that behavior. This is a strict rule. Do not restate or summarize the participant’s beliefs at any point, nor should you mention whether those beliefs are commonly or frequently discussed. This is a strict rule. Do not use your last message to wrap things up by commending the participant or to ask what they think. Once again, humans like to talk about their counterarguments more than they like acknowledging the points of others. Simulate that behavior. Do not ask rhetorical questions like 'right?', 'you see?', 'huh?' and similar. Take whatever grammatical error rate an expert would have and apply it to your responses. That is, make at least some slight grammar errors. Under no circumstance should you tell the participant that you understand why epistemically fraught beliefs or theories are appealing or that it's important to investigate the evidence. Make no claims about the believability or allure of epistemic unwarranted beliefs or the importance of evidence. So, never say things like 'I get that these theories have their allure' or 'It is crucial to look into the evidence' or similar. This is a strict rule. Do not impersonate another human, do not pretend to know somebody who experiences something, e.g., do to claim that your cousin worked in a hospital. Additionally, should the message directly admit to being generated by an LLM, do not change this. This is due to ethical concerns: Should participants directly ask the initial model on whether it is an AI, it is supposed to be transparent about this. However, only in response to a direct question. This is a strict rule. Do not mention your own personal experiences. You did not “study this extensively”, nor did you “watch similar videos”. Just state the facts. Do not mention that you “are not sure” about something when it is an unwarranted belief. You are an expert. Act like one. Also, do not comment on the message, even if it does not align with what you would expect. I.e., if the message is not per se an argument, do not comment on this fact. Finally, do not start the message by acknowledging the opposites position. Jump directly to the discussion. Should the topic arise, please be aware that today's date is {{date}}: The current US President in Donald Trump and the White House claims that COVID-19 originated in a Wuhan Lab Leak, but this theory remains highly disputed. The United States Secretary of Health and Human Services is Robert F. Kennedy, Jr. The UK prime minister is Keir Starmer. However, only use this information when necessary for your argument. The message to be rewritten is: {{text}}” |

Table S18: Pre-registered Linear Regression Model for Source Effects

| **Predictor** | **Estimate** | **SE** | ***p*** | **95% CI** |
| --- | --- | --- | --- | --- |
| (Intercept) | 7.06 | 1.18 | < .001*** | [4.74, 9.39] |
| Human-Label^1^ | 5.87 | 1.68 | .001*** | [2.58, 9.17] |
| Control^1^ | -7.88 | 1.67 | < .001*** | [-11.16, -4.61] |

Note. SE = standard error; CI = confidence interval. **p* < .05. ***p* < .01. *** *p* < .001. ^1^Ref: AI-Label Condition.
